# Approaches to ketamine-assisted couple therapy

**DOI:** 10.3389/fpsyg.2026.1843151

**Published:** 2026-06-04

**Authors:** David O. Avruch

**Affiliations:** Department of Sociology, University of Maryland, Baltimore County, MD, United States

**Keywords:** couple therapy, emotionally focused couples therapy, imago relationship therapy, integrative behavioral couple therapy, internal family systems therapy, ketamine, ketamine-assisted psychotherapy

## Abstract

Ketamine-assisted psychotherapy (KAP) is moving from the periphery toward the mainstream of mental healthcare. Research on the use of KAP in the context of couple therapy, however, remains limited. This phenomenological analysis sought data from psychotherapists (*n* = 9), representing four different modalities of couple therapy, who are incorporating ketamine into their work with dyads. Qualitative interviews revealed substantial diversity with respect to how and why respondents are using ketamine to facilitate attachment processes. Study respondents described ketamine’s ability to help couples reduce fearfulness, deepen emotional expression, communicate frankly, enhance trust, gain perspective on relational dysfunction, and address issues related to trauma. Strategies around preparation, dosing, and integration were explored. Data also revealed how different modalities of couple therapy conceptualize the use of ketamine in unique ways. This study, though limited due to its small sample size, contributes to the development of the nascent, emergent subfield of psychedelic-assisted couple therapy.

## Introduction

The use of psychedelic substances is moving from the periphery toward the mainstream of clinical mental healthcare. Substances including ketamine, MDMA, psilocybin, LSD, and 5-MeO-DMT - alone and in combination with different modalities of psychotherapy - have been and are being investigated in the treatment of depression, anxiety, PTSD, OCD and other psychiatric conditions and may represent a paradigm shift within psychiatry (Schenberg, 2018). This research has generated a body of literature describing these medicines’ efficacy, limitations, safety and risks. As data accumulates, nascent “best practices” are taking shape across a range of applications but have not yet been formalized. Meanwhile, this work is largely unfolding in the absence of the legal infrastructure necessary to support the broader adoption of psychedelic substances - the majority of which remain illegal at the federal level as a result of the set of social policies known as the War on Drugs.

A notable exception is the synthetic chemical compound ketamine, which is classified as a dissociative anesthetic and was originally designated for use in surgical procedures. For several decades, interest has grown in the off-label, legal use of small doses of ketamine to treat psychiatric conditions, both on its own and as an adjunct to psychotherapy. Among practitioners of ketamine-assisted psychotherapy (KAP), ketamine is regarded as a safe, effective medicine which can have psychedelic properties for some individuals at certain dosages. Research by KAP practitioners has led to the development of evidence-based protocols for treating individual mental health problems which are currently in use in the United States and abroad (see, e.g., Yavi et al., 2022; Walsh et al., 2022; Hyde, 2015).

To date, most research on ketamine-assisted psychotherapy has focused on individuals; a smaller body of data describes its use in groups. Only two journal articles (Khalifian et al., 2024; Cornfield et al., 2024) have focused exclusively on the intersection of KAP and couple therapy. However, the use of ketamine in the dyadic context is expanding, and practitioners representing various modalities of psychotherapy are incorporating ketamine into the work of helping romantic partners to strengthen their attachment bonds. The present study sought to explore this emergent form of KAP through qualitative interviews with therapists (*n* = 9) who use ketamine in their work with couples and who are trained in established approaches to couple therapy: Imago Relationship Therapy (IRT), Emotionally Focused Couple Therapy (EFCT), Integrative Behavioral Couple Therapy (IBCT) and/or Internal Family Systems (IFS). The research questions for this study were:

1. How and why are respondents using ketamine as part of couple therapy?
2. What is the relationship between respondents’ modality of psychotherapy and their approach to KAP with couples?

## Methods

### Design and sample

The study followed a phenomenological approach which sought to understand the activity of ketamine-assisted couple therapy from the perspective of respondents. Phenomenology was identified as the appropriate theoretical orientation because the goal of the research was not to test a particular hypothesis per se, but to gather open-ended data about how and why respondents are using ketamine in couple therapy, as well as what meaning they make of this activity (Giorgi, 1985; Smith et al., 2022).

Inclusion in this study required satisfying two criteria, each having up to two elements. The first criterion was to have practiced within a particular modality of couple therapy for at least 5 years, or/and achieved an advanced level of certification within a modality of couple therapy. The second criterion was to have incorporated ketamine into psychotherapy with at least 20 couples, or/and to have offered formal training to other couple therapists on the topic of incorporating ketamine into couple therapy.

Purposive sampling via direct email outreach, convenience sampling via posting on KAP listservs, and snowball sampling were used to recruit respondents. Inclusive of all methods, hundreds of psychotherapists were invited to participate; ultimately, there were nine individuals who expressed interest and who satisfied both inclusion criteria. All nine enrolled in the study and completed its one required element - a recorded interview with the researcher, about 60 min in length, conducted via Zoom. No respondents dropped out of the study. This study was approved by the Internal Review Board of the University of Maryland, Baltimore County (protocol #1450).

### Respondents

Nine respondents provided data for the study. All respondents elected to forego confidentiality and will be referred to in the findings and discussion sections by their first names alongside their primary modality of couple therapy in parentheses; for instance, Beth (EFCT) and Susan (IRT). Waiving confidentiality may have influenced data collection; it is possible that respondents could have shared different information if their identities were kept confidential. Table 1 describes the respondents, their modalities of couple therapy, and their type of clinical license.

**Table 1:** Research respondents.

Name of respondent	Primary modality of couple therapy	Secondary modality	Type of clinical license
Chandra Khalifian	Integrative Behavioral Couple Therapy (IBCT)	Emotionally Focused Couple Therapy (EFCT)	PhD
Kayla Knopp	IBCT	EFCT	PhD
Dick Schwartz	Internal Family Systems Therapy (IFS)		PhD
Kathryn Rheem	EFCT		LMFT
Beth Jaeger-Skigen	EFCT		LCSW
Josh Gressel	Imago Relationship Therapy (IRT)		PhD
Susan McBride	IRT		RN, RSW
Mark Cornfield	IRT		MD
Jayne Gumpel	Syncretic		LCSW

Two respondents (Kayla and Chandra) described themselves as using a primary and secondary modality of couple therapy; all other respondents except Jayne identified as practicing within a particular modality. Regarding her work with couples, Jayne explained that her approach drew from various lineages: IRT, Gestalt therapy, IFS, and mindfulness meditation. Hence, this respondent will be referred to as: “Jayne (Syncretic).” Jayne’s inclusion in the study reflects her satisfaction of the first inclusion criterion: even though she does not identify as practicing exclusively from the lens of IRT, she has achieved an advanced level of certification within that modality. With respect to credentials, it should be noted that respondent Kathryn also holds a Doctorate in Education (EdD) in addition to her clinical degree of LMFT.

Susan and Mark, who practice as a wife-husband team, no longer serve couples in the role of psychotherapists; rather, they offer multi-day “Ketamine Learning Experiences” to psychotherapists and their spouses as a way to train couple therapists in the use of ketamine via experiential sessions. The data they provided for this study emerged both from their experiences as couple therapists (see also Cornfield et al., 2024) and from conducting the retreat-style Learning Experiences (which also involve ketamine-assisted couple therapy). Most other respondents (Beth, Kathryn, Kayla, Chandra, Josh, and Jayne) offer ketamine-assisted couple therapy through their private psychotherapy practices; two (Jayne and Dick) work in the context of ketamine retreats. The retreat model typically consists of multiple KAP experiences in a group format, often involving group psychotherapy before and after, that take place over the course of several days.

### Reflexivity/positionality statement

This study’s author, like the majority of respondents, is a couple therapist in private practice who offers ketamine-assisted psychotherapy. His primary modality of couple therapy is EFCT, with IFS as a secondary modality. In addition to his training as a social worker (with a clinical license of LCSW-C), he is currently pursuing graduate study in sociology. The author identifies as white, cis-male, Jewish and queer.

The author had preexisting relationships with two respondents, Beth and Kathryn, whom he knew based on their shared interests in EFCT and KAP; it is possible that these relationships influenced the data collection process. He did not know any other respondents prior to the study. The author’s familiarity with two of the modalities under consideration in the present study may have influenced data analysis, in that he felt more comfortable describing and exploring the intersection of KAP with EFCT and IFS. As such, efforts were made to present a balanced analysis that afforded equal weight to IBCT and IRT, as well.

The author’s position as a couple therapist who also offers KAP created both access and bias. This insider status may have facilitated rapport-building with respondents, enhanced their perception of the credibility of the researcher and supported emic and clinically-informed interpretation of the data. Simultaneously, the author’s membership in this small group of early-adopters may have increased the risk of confirmation bias - particularly toward interpreting KAP as promising, clinically coherent, or theoretically meaningful. This risk is amplified by the study’s focus on describing favorable as opposed to adverse or neutral outcomes; as such, subsequent studies should interrogate experiences of KAP with couples which do not support the benefits described here, in order to gain a more complete picture of the effects of this medicine on dyadic functioning.

### Data collection and analytical strategy

Primary data collection took place over the course of one interview with each respondent via Zoom, which were recorded. The interviews were transcribed by the researcher and the transcripts were uploaded to NVivo (RRID: SCR_014802), where coding took place according to the principles of Interpretive Phenomenological Analysis (IPA). This method was selected as best suited to achieve the project’s two goals: documenting the nature of the phenomenon at hand and analyzing respondents’ interpretations of that phenomenon (Smith et al., 2022).

Coding and theme development were conducted solely by the study’s author. Codes were generated inductively in a bottom-up fashion, with the researcher initially identifying several dozen codes from the transcripts. First-pass coding took place within a week of data collection, with recoding and refining of codes happening recursively as new transcripts expanded the data pool. Memo writing was used to facilitate the process of recoding. Themes were developed by grouping codes according to recurrent clinical concepts, intervention structures, and rationales for ketamine use. Because no second coder was used, interrater reliability was not assessed. This represents a key methodological limitation of the study in that using multiple coders could have reduced the risk of confirmation bias. The analysis therefore should be understood as an interpretive account shaped by an individual researcher’s clinical perspective, rather than as a consensus-coded thematic analysis.

Ultimately, 26 codes were grouped under six subordinate themes, which were grouped under two superordinate themes: “How I Do This” and “Why I Do This (in the Way that I Do This)”. With the data thus organized, the researcher, relying on his own experience as a couple therapist, identified the content areas most relevant to direct clinical practice and prioritized data related to these and excluded from further analysis all other data. Data were excluded from the present analysis when they concerned topics outside the study’s clinical focus, including (for example) general commentary on the psychedelic field or respondents’ personal experiences with ketamine and other psychedelic medicines. The final analysis prioritized data concerning rationale, safety, eligibility, dosing structures, preparation, integration, modality-specific applications, and trauma-related uses. This choice reflects the subjective assessment of the study’s author regarding which topics would be most relevant to a general clinical audience, given the limitations of space. Appendix A provides a full matrix of codes and themes. Codes listed in Appendix A in italicized font represent data excluded from analysis based on the study author’s assessment of insufficient relevance.

Next, the researcher composed brief (2-4 pp.) interpretive essays summarizing the respondents’ statements with respect to the identified content areas. Each essay was then shared with its respondent via Google Docs, with respondents invited to provide feedback to the researcher on his analysis of their interview. Five respondents gave the researcher feedback about their interpretive essays; this feedback was then added to the data pool and incorporated into subsequent analysis. A small amount of additional data collection, as well as clarification on data already provided, took place via email exchanges.

Finally, after a full draft of the project was completed, all respondents were offered an opportunity to provide feedback to the researcher on the draft. Three respondents offered the researcher feedback on the project draft, which the researcher used to make edits during the final revision process. The purpose of these forms of member checking was to enhance the credibility, accuracy and validity of the study’s findings (Birt et al., 2016).

## Literature review

### Couple therapy

According to Lebow and Snyder (2022, p. 1360), “Couple therapy has emerged as an important, widely disseminated form of therapy.” The respondents to the present study represent four distinct approaches to the task of couple therapy; these approaches represent several that are currently available in the marketplace. Other models include psychodynamic psychotherapy for couples, Gottman Method Couples Therapy, Psychobiological Approach to Couples Therapy, and cognitive-behavioral couple therapy. Such theoretical diversity reflects a lack of unanimity within the field as to the most effective way to help couples feel close in a reliable way. Nor do all couples receive benefit from talk therapy; data suggests that about one third of couples are not helped by therapy (ibid.), while a substantial percentage who do receive benefit eventually relapse to pretreatment levels of relational distress (Bradbury and Bodenmann, 2020). Briefly outlined below are the four modalities represented by this study’s respondents.

#### Imago relationship therapy (IRT)

Originally developed by Harville Hendrix and Helen LaKelly Hunt in 1980, IRT proposes that individuals select partners who they believe, on an unconscious level, can heal their attachment wounds acquired during childhood (Hendrix, 1988). This model blends elements of psychodynamic psychotherapy, cognitive-behavioral approaches and transactional analysis and makes use of a tool called the Imago Dialog - a structured communication format that consists of mirroring, validation and expressions of empathy (Gehlert et al., 2017).

#### Integrative behavioral couple therapy (IBCT)

Originally developed by Andrew Christensen and Neil Jacobson in the 1990s (Anderson et al., 2018), IBCT conceptualizes distress within romantic relationships using the acronym DEEP: *Differences* between partners, *Emotional* sensitivities/vulnerabilities, *External* circumstances and *Patterns* of interaction (Christensen and Doss, 2017). This model simultaneously promotes understanding and acceptance of spousal differences while supporting behavioral changes in interactive habits (i.e., how partners approach and respond to one another) (Jacobson et al., 2000).

#### Emotionally focused couple therapy (EFCT)

Also known as Emotionally Focused Therapy or EFT, this model was originally developed by Susan Johnson and Leslie Greenberg. EFCT proposes that cycles of distress within romantic relationships arise from partners’ unmet attachment needs (Johnson, 2020). Rooted in attachment theory, EFCT embraces the idea that emotions organize attachment processes. The model seeks to generate “corrective experiences” by tapping into emotional expression and choreographing engaged encounters between partners to build trust and connection (ibid.).

#### Internal family systems therapy (IFS)

Originally developed by Dick Schwartz (who served as a respondent in the present study), IFS proposes that the mind is a “plural entity” composed of numerous subpersonalities, known as Parts, and that all individuals hold innate healing energy, known as Self (Schwartz and Sweezy, 2021). In IFS couple therapy,^1^ the goal is to help partners’ protective Parts feel safe enough to relax and step back, allowing the connective energy of Self to emerge and flow organically between the participants (Herbine-Blank et al., 2015). Other interventions may include the therapist working with one partner’s wounded Parts while the other partner observes.

### Psychedelic-assisted psychotherapy (PAP)

The word *psychedelic* means “mind-manifesting” and refers to certain molecules’ capacity to temporarily alter individual perception, cognition, emotion and the experience of the body (Nichols, 2016; Carhart-Harris and Goodwin, 2017). Psychedelic experiences are highly variable and are mediated by the type of medicine that is used, dosage, method of administration, environment of use and mindset or intention of the participant. Psychedelic molecules can generate experiences described as mystical, spiritual and transpersonal; can be deeply meaningful; and can lead to improvements in mental health (Barrett and Griffiths, 2018).

With respect to the structure of treatment, most contemporary approaches to PAP follow a common trajectory, regardless of the psychoactive substance used. First, during preparation sessions, the therapist helps the participant understand what to expect from the medicine, set appropriate intentions and expectations, and become oriented to safety procedures and harm reduction strategies. Following preparation comes the session dedicated to the administration of the medicine, also known colloquially as dosing or journeying. The completed medicine session is followed by integration sessions, wherein the participant and psychotherapist work to make sense of what was revealed during the medicine session, and how to apply any potential insights to the participant’s daily life (Brennan and Belser, 2022). Integration is not limited to psychotherapy; other forms of integration can include journaling, meditation, artmaking, spending time in nature, and other spiritual practices.

As the clinical encounters are different in PAP, so the role of psychotherapist is constructed differently; therapists are often referred to as “guides” whose task is to bring the participant into contact with their “inner healing intelligence” with the assistance of the psychedelic medicine (Aday et al., 2023; Mithofer, 2017). While retaining some traditional elements of psychotherapy (e.g., empathy and nonjudgment), the psychedelic-assisted psychotherapist is often encouraged to use a nondirective approach and to allow the interaction between the participant’s psyche and the medicine lead the way during the dosing session (Cavarra et al., 2022). Various programs have been established to train providers in the principles of PAP; the field is not standardized and encompasses a wide range of approaches (Argento et al., 2024; Phelps, 2017).

Dosage ranges depend on the substance used and the level of intensity desired. Typically, a psychedelic or macro-dose refers to an amount of medicine sufficient to induce a full psychedelic experience; a psycholytic or mezzo-dose describes a lesser amount - not enough to create a psychedelic experience, but sufficient to catalyze the lowering of psychological defenses in order to facilitate psychotherapy; and a micro-dose refers to an amount of medicine that is sub-perceptual. While dosing conventions exist for various psychedelic medicines, these are not formalized, nor are there agreed-upon definitions for what counts as a macro vs. mezzo vs. micro-dose (Cornfield et al., 2024). This reflects the marginality and relative newness of PAP within the western mental health landscape: with one exception (cited below), psychedelic medicines have not completed the Food and Drug Administration (FDA) approval process, which would require the development of recommended dosage and administration schedules.

### Ketamine-assisted psychotherapy (KAP)

One notable exception to the legal limitations on psychedelics is the synthetic chemical compound ketamine, which is classified as a dissociative anesthetic and was originally designated for use in surgical procedures. While not universally considered a psychedelic, ketamine can have psychedelic properties for some individuals at certain dosages (Drozdz et al., 2022); Nutt et al. (2025) refer to ketamine as “psychedelic like” and suggest the label “glutamatergic psychedelic” (p.2). Ketamine is on the World Health Organization’s list of Essential Medicines and is commonly used for sedation and pain relief in surgical and emergency contexts, including with vulnerable populations such as children and elders (Priestley et al., 2001; World Health Organization, 2023). For several decades, interest has grown in the legal, off-label use of low-dose ketamine to treat psychiatric conditions, both on its own and as an adjunct to psychotherapy. “Off-label” refers to the practice of prescribing a drug to treat a problem other than those authorized by the FDA for that particular drug. This is commonplace, with a recent study estimating that off-label prescriptions account for between 21 and 32.3% of prescriptions overall (Van Norman, 2023).

Among KAP practitioners, ketamine is regarded as a safe, effective medicine with particular application in treating depressive disorders (Muscat et al., 2021). Its immediate effectiveness (compared to a period weeks for traditional antidepressant medications), as well as anti-suicidal and anti-anhedonic properties, set it apart from traditional pharmaceutical interventions for treating depression (Singh et al., 2016; Kwaśny et al., 2024). Available data suggests it is markedly more effective at treating depression than the substances typically marketed as antidepressants, especially in the short term (Alnefeesi et al., 2022).

Research by practitioners has contributed to the use of ketamine to treat individual mental health problems (e.g., depression, anxiety and posttraumatic stress), in the United States and abroad (Walsh et al., 2022). In 2019, the FDA approved the prescription of esketamine (a ketamine derivative) for treatment-resistant depression - the first non-surgical application since ketamine was approved in the United States in 1970.^2^ Because prescribing ketamine off-label for psychiatric purposes is legal, and because the medicine tends to be inexpensive in its generic form, KAP has attracted attention as an effective and available form of PAP. Other key advantages offered by ketamine include its rapid onset (within minutes), relatively short window of activation (about an hour for intramuscular injection and sublingual lozenge methods), and the fact that there are no known contraindications for concurrently prescribed psychiatric medications (Beaglehole et al., 2024).

In the context of KAP, ketamine can be administered intravenously (IV), through intramuscular injection (IM), or sublingually (SL) using dissolvable lozenges. The dosage range is different for each method of administration and can be set high enough to induce a dissociative trance-like state that is psychedelic in nature, or low enough such that participants retain the capacity to engage in interactive psychotherapy concurrent with the drug’s activity in their system. For IM and SL applications, ketamine is typically dosed based on the patient’s weight. Ketamine can be administered in a clinic setting (typical for IV, IM and SL), in a psychotherapist’s office (typical for SL) or in the client’s home (typical for SL). When participants ingest a psychedelic dose of ketamine, it is common practice to spend the hour lying down, wearing light-blocking eyeshades and listening to a curated playlist that guides them through their inward-focused journey (Richards, 2017; Barrett et al., 2017).

With respect to contraindications and safety considerations, foremost is the reality that ketamine, like all medicines that relieve physical or psychological pain, can be habit-forming and is a drug of abuse for some individuals (Strous et al., 2022). On the one hand, psychedelic medicines, including ketamine, are currently used in clinical practice to assist individuals seeking recovery from substance use disorders; nevertheless, extreme caution must be exercised when clients present with histories of imbalanced relationships to intoxicants. Other commonly cited psychiatric contraindications for KAP include a personal history or active presentation of psychosis or mania, although the scope and rigidity of these exclusions remain subjects of ongoing clinical debate (La Torre et al., 2024).

The physiological risks of KAP are few. The most common adverse reactions to KAP include nausea, vomiting, and agitation (Dore et al., 2019). These typically resolve within a few hours; nausea and vomiting can be prevented with prescription medicines for this purpose. Rarely, some individuals are allergic to ketamine, leading to systemic reactions consistent with anaphylaxis or histamine release. Because ketamine temporarily increases heart rate and blood pressure, uncontrolled hypertension is contraindicated for KAP (Szarmach et al., 2020).

Globally, the data on ketamine as a psychopharmacological agent - while promising - remains incomplete and contested. Claims about efficacy have not been universally reproduced; for example, a recent meta-analysis of five articles (intervention group *n* = 246) found that ketamine and esketamine did not reduce suicidal ideation in patients with treatment-resistant depression compared to placebo (Wang et al., 2024). Further, while ketamine is associated with rapid symptom relief, outcomes data do not yet support the assertion that ketamine’s antidepressant effects can be sustained over longer periods (Singh et al., 2016). Some authors have cautioned that general enthusiasm surrounding psychedelic therapies could outpace the current strength of the evidence base (Yaden et al., 2022). More data collection is needed, particularly about long-term outcomes, for individuals who participate in KAP.

### Psychedelic-assisted couple therapy and ketamine-assisted couple therapy

Much epidemiological research on the use of psychoactive substances in the context of romantic relationships has focused on the negative effects of use, pathologizing relationships where substance use occurs and minimizing “complex relationship environments that can also include appropriate, supportive partner behaviors around substance use” (Kane et al., 2024, p. 2). With respect to research on individuals who use MDMA, for instance, “the presumption of relational harm is sometimes built into” the study itself (Anderson et al., 2019, p. 5). Other approaches have been more open to observing the use of substances as a way for couples to experience new forms of closeness; for example, Anderson and colleagues have explored how MDMA can assist couples to “express what they are feeling and thinking as well as share more fully in each other’s emotional experience” (2018, p. 16).

With respect to psychedelic-assisted couple therapy per se (as opposed to the therapeutic impact of psychedelic substances used by couples on their own), little research has been published to date. Monson et al. (2020) conducted research into the use of MDMA-assisted Cognitive-Behavioral Conjoint Therapy in the context of treating PTSD, which led to significant improvements in traumatic stress symptomatology. These improvements were sustained in a followup assessment (Wagner et al., 2021).

To date, there have been only two published academic journal articles explicitly focused on ketamine’s psychotherapeutic application to couple therapy. Khalifian et al. (2024) articulated a rationale for incorporating KAP into couple therapy and proposed a framework for the effective use of ketamine with dyads. (Two of that paper’s coauthors, Chandra Khalifian and Kayla Knopp, served as respondents for the present study.) They argue that KAP’s safety and efficacy have been demonstrated through clinical research, and that the use of ketamine facilitates change processes in three key areas of couple therapy: cognition, behavior, and emotion.

Cornfield et al. (2024) have published the only outcomes-focused research on ketamine-assisted couple therapy. (Two of that paper’s coauthors, Mark Cornfield and Susan McBride, also served as respondents for the present study.) Their study enrolled 18 couples, who met weekly as groups of four couples for 4 weeks, to engage in group couple therapy based on Imago Relationship Therapy. These 3-h sessions included 90 min of dyadic work while under the influence of “relational” (i.e., psycholytic or mezzo) doses of ketamine, administered sublingually and augmented as needed with intranasal aerosol ketamine. Quantitative results demonstrated statistically significant improvements in relationship satisfaction following the treatment, while qualitative data explored the wide range of effects produced by ketamine in the context of relationship-focused dialogs, including: “empathogenic effects, mystical/spiritual/psychedelic experiences, [and] anxiolytic and antidepressant effects” (p. 1).

## Findings

The findings of this study are presented in five subsections: *Why Ketamine*?; *Safety & Eligibility*; *Overview of Methodologies*; *Preparation & Integration*; and *Applications for Trauma*. These themes were prioritized by the researcher as most salient for clinical practice. Data not coded to these themes are not presented in this paper, nor are all data coded to these themes included here. Some essential findings are summarized in Table 2.

**Table 2:** Considerations & possibilities for ketamine-assisted couple therapy.

Why KAP	Eligibility	Setting	Timing	Format	Dosage	Interactivity
To soften partners’ defenses	Clients are medically & psychiatrically eligible	Therapist’s office or ketamine clinic	Simultaneous dosing	Sublingual lozenge (clients typically self-administer)	Psychedelic or macro dose	Couple therapy, in person or on Zoom, with one or both partners under influence of ketamine
To lubricate, catalyze, potentiate therapy	Trusting relationship with therapist is present	Clients’ home with therapist present	Back-to-back dosing within the same session	Intramuscular injection (administered by licensed provider)	Psycholytic, mezzo or relational dose	Talk therapy with medicated partner; sober partner observes
Trauma response impeding process	Sufficient non-ketamine couple therapy completed	Clients’ home with sober chaperone	Alternate partner dosing in sequential sessions			Inward-focused journey followed by facilitated couple therapy
Talk therapy is not adequate to serve the couple	Therapeutic container created by retreat setting	Retreat center	Only one partner receives ketamine			Inward-focused journey followed by individual, dyadic or group integration

### Why ketamine?

Respondents endorsed a variety of reasons for incorporating KAP into dyadic psychotherapy; on the most basic level, ketamine’s safety, legality and efficacy were major attractants for all respondents. Some respondents praised KAP’s lower time commitment - usually 2–3 h are set aside for a KAP session - relative to longer-acting psychedelic medicines like psilocybin or MDMA. Several respondents expressed the opinion that KAP is indicated in situations where talk therapy alone is an insufficient intervention to serve the couple.

In terms of its effectiveness for couple therapy, Beth (EFCT) appreciates how ketamine helps to “soften” attachment-related wounds, allowing clients to “touch vulnerability or show vulnerability” in new ways. Indeed, multiple respondents used variations on the word “soften” to describe the effects of ketamine. Kayla (IBCT/EFCT) finds that ketamine can help to reduce clients’ fear response, making it “easier for couples to engage in… core couple therapy processes.” This belief is reflected by Dick (IFS), who, using the language of his modality, observed that ketamine “relaxes protectors so that you can get to [deep] places… in a short time.” Josh (IRT) finds that the medicine “makes it much easier to cross the bridge”. This is IRT language that refers to the process of “leaving your world behind and joining your other person in their world.” Josh expressed his opinion that incorporating ketamine into treatment can reduce the global amount of sessions necessary for couples to achieve their therapeutic goals.

Mark (IRT) believes that ketamine can be a “lubricant” for “any good therapy” by “putting the person in a place of receptivity and being able to see themselves in different ways and take risks… that don’t even feel like risks when you’re on ketamine.” Kathryn (EFCT) described ketamine as a “benevolent disruptor” of traditional relational patterns that “potentiates [and] catalyzes therapy”. For Chandra (IBCT/EFCT), the effects of ketamine can include “more cognitive flexibility, more openness, more empathy”. Susan (IRT) believes that ketamine helps with “taking your mask off”; for her, under the relational dose of ketamine, “defenses are gone, people are more open, people will share things they were too afraid to share [otherwise]”. Jayne (Syncretic) values ketamine in couple therapy because it “opens up a field of awareness that goes beyond where they’re stuck”.

### Safety and eligibility

With respect to safety, Susan (IRT), a trained nurse who works side by side with a medical doctor, states that “we never felt there was any medical risk at all.” That said, respondents affirmed the importance of respecting traditional contraindications for KAP, such as active substance misuse. In terms of contraindications at the level of the dyad, Kathryn (EFCT) stated that couples should possess sufficient trust to be able to display emotional vulnerability with one another, and that the therapist must assess whether both partners are interested in pursuing KAP (as opposed to one member of the dyad exerting pressure on the other). Kayla (IBCT/EFCT) observed that the traditional contraindications for couple therapy also must be observed; i.e., screening out couples who are dealing with intimate partner violence, patterns of coercion and control, and “anything that speaks to a lack of safety inside the relationship.”

Respondents named other safety measures as well. Dick (IFS) believes that individuals who engage with KAP in the context of a short-term retreat setting should have an ongoing psychotherapist to continue the work of integrating the ketamine experience afterward. Several respondents require clients to measure and report their heart rate and blood pressure before ingesting ketamine, to ensure that they are within an appropriate range for dosing to take place. Some keep an emesis bucket on hand in case of vomiting. Mark (IRT), a medical doctor, keeps a few “rescue medicines” handy: for high blood pressure, nausea and vomiting, as well as a urinary anesthetic (for mild irritation of the lining of the bladder, which can occur occasionally with ketamine). Clients are advised not to drive after a KAP session, and to have rides prearranged. No respondent reported any medical emergencies arising from their experiences with KAP.

Three respondents who conduct KAP sessions remotely via Zoom (Kathryn [EFCT], Kayla and Chandra [both IBCT/EFCT]) require clients to have a “sober chaperone” in their home during dosing. Chandra described how, with couples who are new to ketamine or psychedelics, she will be present for the entirety of their first dose. For subsequent doses, at her clinical discretion, she may offer clients the option for self-guided sessions with a sober chaperone present. In such situations, Chandra may connect with the couple on Zoom before and/or after the medicine session; or, they may dose on their own and then reconnect with the therapist at their next scheduled non-medicine integration session. For these types of journeys, Chandra offers the couple a curated playlist to listen to during the journey as well as prompts for journaling or verbal interaction after they emerge. Kathryn’s method is to set up a text message thread for herself, the clients and the chaperone, and have the chaperone advise her via text when the clients ingest the medicine and emerge from their journey. One advantage to this method is that it increases access to KAP by reducing the price, given that the therapist’s hourly rate is typically the most expensive element of the treatment.

### Overview of methodologies

Nearly every respondent described different ways of “doing” KAP with couples; several respondents endorsed the validity of multiple approaches. Some continue to experiment over time with different ways of deploying ketamine in order to facilitate couple therapy; others have maintained a consistent approach. This subsection seeks to delineate some primary options described by respondents.

A consistent finding is the use of sublingual (SL) lozenges as the method of administration for working with couples. Mark (IRT) was the only respondent who is licensed to prescribe medication; all other respondents collaborated with trusted prescribers who conduct independent evaluations of clients prior to making ketamine available. For KAP that takes place in private practice, the prescriber will typically have a compounding pharmacy produce the lozenges and send them by mail to the client’s home address. Then, at the time of the KAP session, the clients will self-administer the medicine either in their home or in the therapist’s office. In the setting of a multi-day KAP retreat, the prescribed ketamine is dispensed by a licensed healthcare professional. Some retreats may also offer ketamine via intramuscular injection.

Substantial variation in dosage ranges was reported for SL ketamine; according to respondents, the range for a relational or psycholytic dose is between 50–150 mg, while a macro or psychedelic dose is between 200–400 mg. These ranges reflect diversity in how different bodies metabolize the medicine, and thus how intensely the ketamine is experienced. For this reason, Jayne (Syncretic) refers to an individual’s first experience with KAP as the “calibration dose” - a chance to become aware of how the medicine impacts the body at a particular dose. Regardless of the dose, the effects of SL ketamine are felt for about 60 min, at which point the client begins to emerge from the non-ordinary state of consciousness.

Multiple respondents described dosing as a collaborative endeavor between the client, the therapist and the prescriber. Indeed, there are various reasons that respondents may use psychedelic vs. psycholytic dosing. Kayla and Chandra (both IBCT/EFCT) rely primarily on simultaneous dosing at the psychedelic level. This involves an inward-focused journey, with the client wearing light-blocking eyeshades and listening to a curated playlist on headphones for about 60 min. Chandra appreciates how the dissociative properties of a macro dose of ketamine can pull clients out of typical patterns of thought and emotion; she believes this experience can help couples gain a new perspective on their dysfunctional pattern of interaction.

Mark, Susan, and Josh (all IRT) rely on simultaneous psycholytic SL dosing. In other words, both members of the couple will ingest the medicine at a dosage that allows them to interact with one another and the therapist, as opposed to entering the dissociative, psychedelic, inward-focused state induced by a macro dose. Josh conducts traditional IRT with his couples while they are under the influence of ketamine. Susan and Mark, a wife-husband team who work with groups of four to five couples at a time, set up their clients to interact with one another while under the influence of the medicine. The couples respond to conversation prompts from a manualized protocol, rooted in IRT, which Mark and Susan developed for this purpose. During the medicine session, Mark and Susan will circulate and monitor the dyads, but usually do not facilitate their interactions directly. They also have offered “booster” doses as needed via intranasal aerosol ketamine, in order to keep clients “at the desired relational level” for similar lengths of time. This is helpful due to diversity with respect to how quickly different bodies metabolize ketamine.

Jayne (Syncretic) offers KAP to couples in her private practice, and also works with groups of couples in the setting of multi-day KAP retreats. There, over the course of three KAP sessions, she will have couples complete inward-focused journeys with eyeshades and headphones, with the option to experiment with different dosages within a set range. For Jayne (and other respondents), an inward-focused journey can be therapeutic at lower as well as higher doses of ketamine. Emerging from the medicine, Jayne’s couples will have the option to engage with one another using the Imago dialog; however, the individual members of the couple may have different timelines in terms of how their body has metabolized the medicine, which means they may not be available to engage or feel ready to speak at the same time. Jayne explained, “If they’re not in alignment… the person who’s coming out might just hold the other person, or hold his or her or their hand. Or… just take their eyeshades off and just gaze [at their partner]… There’s no set rules about it. You’re working with what’s occurring.”

Kathryn (EFCT) typically works with psycholytic, non-simultaneous dosing. She had originally expected to facilitate couple therapy with both partners under the influence of ketamine at the same time, but found that because people metabolize ketamine at different rates, simultaneous dosing sessions were unpredictable. Over time she developed a back-to-back dosing structure, with one partner ingesting the ketamine 30 min after their partner. Then, Kathryn waits until after the medicine has “peaked” for the second person before she initiates psychotherapy. At this time, she explained, “the window is open” for psychotherapeutic engagement. Sometimes this looks like traditional EFCT, i.e., addressing barriers to attachment by engaging with emotion. Other times it means following “the lead of the medicine” in terms of what content is explored. Kathryn’s other method for non-simultaneous dosing is to have one partner ingest the medicine in one session, then the other partner ingest the medicine during the next session. For Kathryn, this method is “perhaps… ideal.” Because ketamine often amplifies primary emotion, it increases vulnerability; Kathryn believes that when this risk is structured safely - i.e., in a mutual and reciprocal way - it builds trust between partners.

Beth (EFCT) typically starts with each member of the couple receiving the medicine on different days. She offers the option of either a psychedelic or psycholytic dose, but finds that most clients opt for a psychedelic dose for their first experience. Beth also lets the person receiving the medicine decide whether their partner should be in the office observing them, or outside the office but “on call” to come in if desired. “Often, I’ll ask halfway through,” she stated. For Beth, when a partner who is outside the room gets invited in to see their partner in the medicine, this can be a powerful opportunity for connection because “you’re so lovable when you’re in that vulnerable state.” With psycholytic dosing, if the client chooses for their partner to be present, Beth may conduct EFCT if it feels right for the couple. Once both partners have gained familiarity with ketamine and identified an appropriate psycholytic dose, simultaneous dosing can take place, during which Beth will conduct EFCT. She described the experience of conducting couple therapy under these conditions as feeling “like butter.”

Dick (IFS) has conducted couple therapy with ketamine in two different ways. One option is to have one member of the couple take a psychedelic dose of ketamine while their unmedicated partner bears witness/holds space. Dick explains that, once the medicated individual has completed their inward-focused journey and exited the non-ordinary state of consciousness, they emerge into a “golden hour” where “there’s just a huge amount of Self present”. In this context, the unmedicated partner will observe Dick conducting individual psychotherapy with the post-medicine partner, focused explicitly on healing that person’s vulnerable/exiled Parts. Dick believes that, in addition to offering healing to the person receiving therapy, observing one’s partner in this vulnerable, open, post-medicine state can have a profound effect on the unmedicated partner, whose “protectors are also off-line so their heart’s very open and they can express a lot of love”. In other words, whereas outside the context of ketamine-assisted couple therapy the couple might be accustomed to approaching one another from a state of emotional protectedness, the ketamine will “put [the individual’s] protectors to sleep” - which can evoke a comparable dropping of armor from the observing, unmedicated spouse.

Another option for Dick is to offer simultaneous dosing at the psycholytic level. Within this version, therapy can look a few different ways. One way is for the therapist to invite the partners to engage directly with one another while in the medicine, with the therapist’s assistance if protective Parts emerge. Within this model, attachment experiences emerge from the partners being able to speak to one another from a place of sufficient Self energy, as well as from observing one another interact with their own protective Parts. Another version of simultaneous dosing is to have the partners taking turns interacting directly with Dick, who does individually-focused psychotherapy with each person. In this version, attachment experiences emerge from the act of bearing witness to one’s partner in a state of vulnerability and openness as they work in turn to receive healing through the interaction with the therapist.

### Preparation and integration

According to respondents, preparation for KAP for couples has much in common with individual KAP. In particular, respondents named the importance of orienting clients to the medicine by helping them understand its effects, side effects, duration and intensity. Another traditional element of preparing for KAP is setting intentions and managing expectations, as well as attending to the relationship between the therapist and clients. Jayne (Syncretic) described that when working in the context of a retreat setting, she will complete at least one 90-min therapy session in advance with each participating couple. Chandra and Kayla (IBCT/EFCT), following the protocol of IBCT, will complete a minimum of four assessment sessions (two with the couple, one with each member of the couple) prior to bringing ketamine on board. For Beth (EFCT), before incorporating ketamine, the couple will have completed enough therapy to be aware of the negative cycle within their relationship, and each partner’s role in sustaining it.

Other elements of preparation are specific to dyadic work, and/or to the modality of the practitioner. Kayla (IBCT/EFCT) described “attending to the relational frame” in preparatory work; in particular, helping clients articulate how they want the ketamine experience to impact their relationship. Mark and Susan (IRT) make sure that, prior to using ketamine, clients are “comfortable in the Imago process”. (In the work described in the paper published in 2024, Mark and Susan included only couples who had already completed one of their weekend-length Imago workshops.) Kathryn (EFCT) talks with clients about balancing the open-ended, nonlinear, and unpredictable nature of a KAP experience with the focused, directive style of her modality. Dick (IFS) advises clients that the medicine may invite them into a state of emotional vulnerability that they do not typically access during waking life, as protective Parts temporarily go “offline.” Several respondents identified working with clients to address concerns they have about being emotionally open with their partners as a key element of preparation for KAP in couple therapy.

Integration refers to the non-medicated experiences, including but not limited to psychotherapy, that follow the KAP session. Integration was identified by multiple respondents as crucial to receiving benefit from ketamine, but there was diversity with respect to when respondents believed integration ought to take place. Some, like Beth (EFCT), facilitate integration immediately after the client emerges from the psychedelic ketamine experience (with or without their partner present). Some respondents who work with groups of couples in retreat settings (Susan and Mark [IRT], Jayne [Syncretic]) engage in multi-couple integration processes, so that clients can learn from the experiences of other couples. Kayla and Chandra (IBCT/EFCT), on the other hand, typically do not facilitate integration immediately after the clients emerge from the ketamine. Kayla will encourage the partners, after emerging from the non-ordinary state of consciousness, to share about their experience using “soft prompts.” Her goal is to have partners “talk about [the psychedelic journey] as a way of consolidating it in memory”. For Chandra, it is ideal to schedule an integration session for the following day, and focus on how to integrate the information revealed during the medicine session into the couple’s relationship. Kathryn (EFCT) is flexible in how she engages integration: some couples may alternate between KAP and non-KAP sessions, others may do several KAP sessions in a row.

### Applications for trauma

Respondents endorsed the notion of ketamine-assisted couple therapy as an appropriate intervention to treat psychological distress created by trauma exposure, which several noted can substantially impede the couple therapy process if unaddressed. Beth (EFCT) expressed her belief that ketamine can facilitate therapy in situations where one or both partners have experienced trauma very early in life, stating that KAP “generates more empathy, compassion and connectivity” around trauma. She also observes how ketamine can help clients access trauma-related content that is “unconscious” or “blocked.” Josh (IRT) believes that using KAP with couples in the context of trauma “strengthens the relationship by showing the healing power of being vulnerable in relationship”. In other words, for Josh, healing from traumatic stress in the context of couple therapy builds the attachment bond, and ketamine can facilitate that process. For Jayne (Syncretic), “ketamine has a way of cutting through” the cycles that couples can get locked into that are connected to an “outsized response” that one partner may evince as a result of trauma exposure. She believes that greater empathy can emerge as a result of the “distance” or dissociation ketamine provides - not only from the memories of traumatic events, but the beliefs and narratives about self and others that emerge as a result of exposure to trauma. Kayla (IBCT/EFCT), while endorsing KAP for this purpose, cautions that it is important to use a couple therapy modality that is appropriate for addressing trauma in the relationship, as well as for the therapist to know how to treat trauma in a relational context.

## Discussion

The findings of the present study help to flesh out the emergent practice of ketamine-assisted couple therapy. It is not known how many providers are currently offering this service in the United States; however, this number is probably small, but will grow as the use of ketamine continues to move toward the mainstream of psychotherapy and as rates of therapy utilization continue to increase over time (Lebow and Snyder, 2022).

The most prominent finding from this study was the diversity of approaches to the task of ketamine-assisted couple therapy. This finding can be interpreted in several ways. First, ketamine-assisted couple therapy encompasses multiple styles which coexist in the marketplace of ideas. Next, ketamine is appreciated by study respondents as a safe, versatile and efficacious medicine when used appropriately. Third, both psychedelic and psycholytic dosages are appropriate for psychotherapy with dyads, for different reasons. And last, that the traditional PAP model (preparation → dosing → integration) appears to work well when applied to ketamine-assisted couple therapy.

With respect to the first observation, we can locate psychedelic-assisted couple therapy within the established subfield of traditional couple therapy. Like individual psychotherapy, couple therapy currently houses various theoretical orientations, each with its own set of technical interventions. Indeed, forms of psychotherapy, rather than being winnowed down over time toward a “most effective” modality, continue to flower into an expansive menu of treatment options. In a basic sense, ketamine-assisted couple therapy represents the continuation of that sociomedical process. i.e., it is the next way, a new way, that couple therapy is being practiced; it will not be the last way. Based on the history of psychotherapy, it appears logical to deduce that as long as the demand for therapy grows, new approaches will continue to emerge.

Nevertheless, respondents in the present study believe that KAP, when indicated, represents a step forward in their quest to help couples - which is why they take the trouble to incorporate this medicine into their work. As the findings demonstrate, facilitating KAP in the dyadic context is no small feat and requires training, collaboration, patience, vigilance and a tolerance for exposure to the unknown. Significant care is taken by practitioners to facilitate clients’ safe, productive medicine experiences, but the outcome of that experience is not known in advance. Kathryn’s (EFCT) description of ketamine as a “benevolent disruptor” reflects other assessments of this medicine as a nonspecific amplifier of psychological and emotional energies and processes.

Indeed, the present findings affirm that ketamine does not offer a straightforward, point-and-click experience for either the couple or their therapist. Rather, engagement with ketamine (as with all psychedelic medicines) represents a deliberate encounter with what lies beneath the surface of human consciousness. The therapist’s task is to facilitate this encounter, receive what emerges, and incorporate that content into the work of couple therapy; this study’s respondents believe strongly that the benefits of this activity outweigh its downsides and risks. Therefore, another conclusion to be drawn from the diversity of findings is that when it comes to ketamine-assisted couple therapy, at present it appears that multiple valid approaches exist. Hence, the answers to this study’s first research question - How and why are respondents using ketamine as part of couple therapy? - appear to be: in various ways, because they believe ketamine works.

With respect to the second research question - What is the relationship between respondents’ modality of psychotherapy and their approach to KAP with couples? — the findings offer some ideas.

### IRT

The practitioners of Imago Relationship Therapy (Josh, Mark, Susan) rely on simultaneous, psycholytic dosing with the goal of having the members of the couple interact with one another while under the influence of ketamine. For them, the effects of the mezzo dose of ketamine produce conditions where “crossing the bridge” can take place in a profound way. Jayne, whose style includes IRT principles and techniques, utilizes both psychedelic and psycholytic dosages, and generally invites clients to complete inward-focused journeys during the window that the medicine is most active in their systems. For Jayne, Susan and Mark, conducting integration in the context of a larger group of couples offers particular benefits.

### IBCT

Chandra and Kayla, who practice Integrative Behavioral Couple Therapy, primarily utilize simultaneous, psychedelic dosing with inward-focused journeys. For them, the impact of KAP on couple therapy unfolds most of all during the non-medicine integration sessions, which are ideally scheduled within a day or two of the ketamine journey. Their approach to couple therapy conceptualizes an inability to accept one’s own and one’s partner’s experience as a key driver of relational dysfunction; for Kayla and Chandra, part of ketamine’s power lies in its ability to invite dyads into a fuller experience of human emotion. This expansion of awareness occurs via the psychedelic journey, and is applied to the relationship during integration.

### EFCT

The respondents who practice EFCT, Beth and Kathryn, each described ways of interacting with the medicine in terms of dosage and timing that produced outcomes they viewed as therapeutic. Within their modality, evoking emotion is viewed as a central component of the couple therapy process; both practitioners value ketamine for its capacity to amplify primary emotion. Further, EFCT’s focus on helping partners access vulnerability in order to generate emotionally corrective experience is reflected in how Kathryn and Beth conceptualize the non-simultaneous use of ketamine: Beth describes the emotional connection that comes from the sober member of the couple observing their spouse in the medicated, vulnerable state; Katherine respects that receiving ketamine while your partner remains sober can feel emotionally risky, and that exposure to this risk, when structured in a balanced way by the therapist, can facilitate attachment.

### IFS

Dick’s conceptualization of ketamine-assisted couple therapy clearly reflects the core tenets of the modality he developed. IFS proposes that if protective Parts can create space, then the wounded or exiled Parts underneath can be healed via psychotherapy; and, if wounded Parts experience healing, clients become more balanced psychoemotionally and better able to relate to themselves and their spouses. Additionally, in the context of couple therapy, attachment is furthered by one partner observing their spouse’s process of healing. Ketamine, in Dick’s view, facilitates this process substantially by “[putting] protectors to sleep” and generating “a huge amount of Self [energy]”. He described multiple approaches to ketamine dosage and timing that effectuate these outcomes.

Within this set of answers to the second research question, it can be observed how each modality of couple therapy conceptualizes the purposes of ketamine in slightly different ways, according to its unique perspective on the work of facilitating dyadic attachment. This adaptability speaks to Mark’s observation that ketamine can be a “lubricant” for “any good therapy”. Nevertheless, given that ketamine-assisted couple therapy is still emerging as a form of clinical practice, it is too early to state with certainty whether different modalities of couple therapy lend themselves to particular formats of ketamine-assisted couple therapy. It seems likely that other practitioners within each of these four styles - as well as from other approaches to couple therapy, not studied here - will contribute to the development of new models for how to use ketamine to help partners feel close to one another.

## Limitations

The present findings should be interpreted in light of substantial limitations in sampling, selection and analysis. First, the study reported data from only 9 respondents. All respondents were highly specialized clinicians operating at advanced levels of clinical practice, as well as early adopters of ketamine-assisted psychotherapy with couples. As such, they are likely to be more favorably disposed toward KAP than clinicians who have chosen not to incorporate ketamine into couple therapy, clinicians who have discontinued the practice, or clients who experienced limited benefit or harm. Hence, conclusions cannot be drawn from this study about the efficacy, safety, or generalizability of ketamine-assisted couple therapy. Instead, the study describes how a small group of experienced practitioners are conceptualizing and implementing an emerging intervention. The small sample size also limits generalizability with respect to two key issues: representativeness of modality of couple therapy and the practice of KAP. Psychotherapy is both art and science; different practitioners bring different skills to the same task. It is not necessarily the case that the respondents to the present study embody all essential tenets of their respective modality, or of KAP; rather, it makes more sense to observe them as inhabiting their affiliated modality, and the world of ketamine-assisted psychotherapy. In other words, this study’s respondents are pioneers who are actively merging fields of clinical practice. Hence, the information they offer should be received as descriptive as opposed to definitive.

A related limitation concerns potential reporting bias. Respondents’ accounts of using ketamine with couples were largely positive, and the study did not systematically capture failed cases, client dissatisfaction, dropouts, adverse psychological reactions, or relational deterioration following KAP. Although respondents discussed screening procedures, contraindications, variability of response, and safety measures, the absence of substantial negative case material limits the conclusions that can be drawn. Future research should include adverse event reporting, and perspectives from clinicians who have chosen not to use or to discontinue KAP with couples. No respondents in the present study reported medical emergencies in their use of KAP with couples; however, this absence should not be interpreted as evidence of safety, given the study’s design and sampling limitations.

Additionally, the limitations of space necessitated omission of collected data regarding the practice of KAP with couples; a fuller picture of this evolving practice will continue to be painted as the literature expands (See the Methods section for a discussion of excluded data.) Next, the study did not focus on diversity among couples: the specific concerns of racial minority couples, interracial couples, LGBTQ+ couples and low-income couples were not addressed. The stressors faced by these couples are unique (see, e.g., Lavner et al., 2018; Irby-Shasanmi and Erving, 2022; Rostosky and Riggle, 2017; Karney, 2021); hence, the need for effective and culturally appropriate interventions may be greater. The only exception to this limitation concerns the discussion of using sober chaperones as a way to reduce the price of ketamine-assisted couple therapy. Additionally, the complexities brought by couples who engage in polyamory and other forms of nonmonogamy were not addressed in the present study.

Finally, because this study focused on clinicians, both outcome data and client perspectives were absent from analysis. These data are required in order to comprehensively assess the efficacy and feasibility of KAP with couples. Hence, the findings of the present study, while largely favorable, cannot be interpreted as sufficient evidence that the practice under examination merits broader acceptance. It should be again emphasized that the findings are not intended to be conclusive regarding how this work ought to unfold; rather, these data are descriptive of some of the options currently being explored by competent clinicians. It is expected that this sub-subfield will continue to evolve over time.

## Future research

There is a clear need for further research into the practice of ketamine-assisted couple therapy. As this form of clinical practice moves toward the mainstream, more outcomes data should be gathered in order to formally assess the benefits and risks of this approach to dyadic psychotherapy. Though comparative analysis regarding different modalities of psychotherapy may become possible as the data pool expands, the more important first task is to compare the effectiveness of ketamine-assisted couple therapy with traditional couple therapy - especially with respect to durability of the effects of treatment. Josh’s belief that the incorporation of ketamine may reduce the number of sessions needed for a couple to achieve their therapeutic goals is, if true, important. Especially for couple therapy that takes place on a fee-for-service basis outside of insurance networks, developing methods to streamline the process will have direct benefit for couples. Additionally, researchers affiliated with particular modalities of couple therapy may seek to further cultivate and refine approaches to KAP that align philosophically and methodologically with their respective schools of thought.

## Conclusion

This study’s findings reveal that globally, there is substantial diversity with respect to how and why respondents are incorporating ketamine into their work with couples. As with individual KAP, “best practices” have not yet been formalized; however, various protocols for different use-cases are actively being developed by clinicians and their clients through iteration and experimentation. Such open-endedness speaks to ketamine’s versatility as a psychopharmacological agent, as well as a shared belief among practitioners that ketamine is safe and efficacious in psychotherapy when used appropriately. The data from this study contribute to the growing awareness that, when ketamine is deployed thoughtfully, there are multiple ways to conduct this form of therapy that will produce outcomes beneficial to clients. Future research may confirm that this assertion applies to working with couples as well as individuals.

## Data Availability

The raw data supporting the conclusions of this article will be made available by the authors, without undue reservation.

## Footnotes

1 Couple therapy in IFS is also known by its own name, Intimacy From the Inside Out (IFIO). However, because the respondent for the present study described his work with couples as IFS as opposed to IFIO, the term IFS will be used in this paper.

2 Marketed under the brand name Spravato in the form of a nasal spray, esketamine is distinct from generic ketamine, which is the focus of this article - though esketamine may also have application for couple therapy.

## Appendix

**Table A1:** Codes and themes.

Superordinate theme	Subtheme	Code
How I Do This	Basic Logistics	*Discussing KAP with Clients*
		Safety Protocols
		Preparation
		Integration
		Set & Setting
		Remote vs. In-Person
	Advanced Logistics	Eligibility Considerations
		Dosing & Prescribing
		Risks & Adverse Events
		Trauma Applications
		Methods of Administration
	How to Do KAP with Couples	Methodology of KAP for Couples
		Anecdotes from Sessions
	Practice Info	*Client Demographics*
		*Money/Fees*
		*Marketing/Advertising*
Why I Do This (in the Way That I Do This)	Relationship to KAP & PAP	*How I Got into KAP/PAP*
		Why KAP
		*Why PAP*
		*Personal Experiences with Psychedelics*
		Provider’s Experience Level
		*Other Psychedelic Medicines*
	Modality, Training & Experience	My Modality’s Methodology
		*Opinions about Couple Therapy*
		Theory of Modality
		My Style & Training in Couple Therapy

Codes presented in italic font represent collected data excluded from analysis.
